# Improvement in sensitivity for lateral flow immunoassay of ferritin using novel device design based on gold-enhanced gold nanoparticles

**DOI:** 10.1038/s41598-022-11732-5

**Published:** 2022-05-12

**Authors:** Pattarachaya Preechakasedkit, Kanyapat Teekayupak, Daniel Citterio, Nipapan Ruecha

**Affiliations:** 1 Metallurgy and Materials Science Research Institute, Chulalongkorn University, Soi Chula 12, Phayathai Rd., Pathumwan, Bangkok, 10330 Thailand; 2 Electrochemistry and Optical Spectroscopy Center of Excellence (EOSCE), Department of Chemistry, Faculty of Science, Chulalongkorn University, Pathumwan, Bangkok, 10330 Thailand; 3 Department of Applied Chemistry, Faculty of Science and Technology, Keio University, Yokohama, Kanagawa 223-8522 Japan

**Keywords:** Biomarkers, Analytical chemistry, Screening

## Abstract

This work introduces a low-cost adhesive tape combined with a hydroxylamine/polyvinyl alcohol/polyethylene oxide (HA/PVA/PEO) blend film to fabricate novel devices for improving sensitivity of gold nanoparticle (AuNP)-based lateral flow immunoassays (LFIAs) via two platforms: (1) LFIA device with integrated gold enhancement and (2) LFIA device with two independent sample inlets. The detection of ferritin has been used for proof-of-concept. The adhesive tape inserted in the devices assists to separate two solutions independently flowing from two different inlets toward a nitrocellulose membrane. On-device gold enhancement was achieved by the enlargement of AuNPs via the catalytic reaction of KAuCl_4_ and HA using the HA/PVA/PEO blend film easily prepared via a solution-casting technique, which could delay the flow of HA released from the film for 180s and improve storage stability of the device. Under optimal conditions evaluated by naked eyes, the gold enhancement (LOD = 0.5 ng/mL) and double-sample inlet (LOD = 2 ng/mL) devices exhibited 20-fold and fivefold higher sensitivity respectively than a conventional device, verifying the sensitivity improvement. Furthermore, the proposed device was successfully detected ferritin in human serum samples within 10 min via naked-eye observation, exhibiting rapidity and simplicity of use, and the capability to perform on-site assays.

## Introduction

Lateral flow immunoassays (LFIAs) based on gold nanoparticle (AuNP)-labeling are widely used as an alternative method for point-of-care testing (POCT) diagnostic tools^1^ because of many advantages, such as lower cost, shorter analysis time, simpler process, portability, capability to conduct on-field measurements and minimal requirement of reagents^2,3^. However, the AuNP-based LFIAs conventionally suffer from a relatively low analytical sensitivity^2,4^. Normally, the detection sensitivity of a colorimetric sandwich immunoassay depends on the binding capability of the target analyte with the labeled antibody and the capture antibody immobilized at the test line^5^. The possibility of increasing the probability of binding between the target analyte and the two antibodies or the amplification of the colorimetric signal would enhance the detection sensitivity. Therefore, there have been various methods developed for the sensitivity improvement of the colorimetric signal based on AuNP-labeling, including solution flow rate control and signal amplification using nanocomposites of AuNPs and other labels, such as AuNPs/enzyme^6,7^, AuNPs/gold^8,9^, AuNPs/silver^10,11^, AuNPs/platinum^12^, AuNPs/fluorophore^13,14^ and hollow nanogold microspheres (HGMS)^15^. Controlling the solution flow rate aims at increasing the interaction time for binding of the target analyte and the labeled antibody by delaying the flow or temporarily accumulating solutions. This has been achieved through various LFIA device modifications, including wax-printed pillars^16^, incorporation of polydimethylsiloxane (PDMS) into the test strip^17^, dissoluble saline modified nitrocellulose membranes (NCM)^18^, cotton threads embedded on the NCM^19^ and soluble wax barriers located downstream of a test line for the temporary accumulation of solution^20^.

Interestingly, Zhang et al.^21^ developed a device based on a stacking flow immunoassay for overcoming the particle aggregation and adhesion on the device when high concentrations of proteins in salivary fluids were detected. A liquid permeable film originally applied for a polymerase chain reaction (PCR) plate was utilized as a flow regulator to guide the sample and the labeled antibody to the test strip through different paths. This device could solve molecule accumulation and adhesion in high protein concentrations. However, it still suffered from disadvantages, such as low sensitivity and the use of an expensive and rare liquid permeable film.

In the past decade, polymer blend films using polyvinyl chloride (PVA) and polyethylene oxide (PEO) as polymer carriers complexed with alkaline salts have been widely employed to form a polymer electrolyte film for the development of batteries, fuel cells and electrochemical sensors^22,23^. The use of a polymer electrolyte film provides numerous advantages, such as easy fabrication of thin films with the desired sizes, thermal stability, long lifetime, and tensile strength^24^. PVA and PEO are non-toxic and biodegradable hydrophilic polymers, which can be easily formed into films via the solution-casting method^23^.

This work proposes a method for sensitivity improvement of the AuNP-based LFIA using an extremely low-cost adhesive tape easily purchased from local markets and a HA/PVA/PEO (hydroxylamine: HA) blend film prepared via a solution-casting technique to fabricate a novel device applied to the detection of ferritin as a model analyte. Ferritin is an important clinical biomarker for iron status assessment, and ferritin levels in serum closely correspond to the total levels of iron storage in the human body^25–27^. The improvement of the assay sensitivity on the device occurs by an independent double-sample inlet and signal amplification using gold enhancer solution composed of KAuCl_4_ and HA, a reducing agent. The role of the adhesive tape within the device is to separate the two solutions independently flowing from two different inlet pads. The HA/PVA/PEO blend film serves the purpose of delaying the release of the HA reducing agent. Parameters influencing the device fabrication and performance were systematically investigated, including the concentration of the antibodies, volume of the labeled antibody, blocking condition of the device and concentration of the gold enhancer. Furthermore, the analytical performance was evaluated by a qualitative analysis through a simple naked-eye observation and a quantitative analysis using a smartphone camera and subsequent data analysis. Finally, the developed device was successfully applied for the analysis of human serum samples, demonstrating its usefulness for early screening, and monitoring of iron deficiency anemia as well as its high practical applicability potential for detection of other biomarkers in further studies.

## Results and discussion

### Separation of solution flow using a low-cost adhesive tape

**Figure 1:**
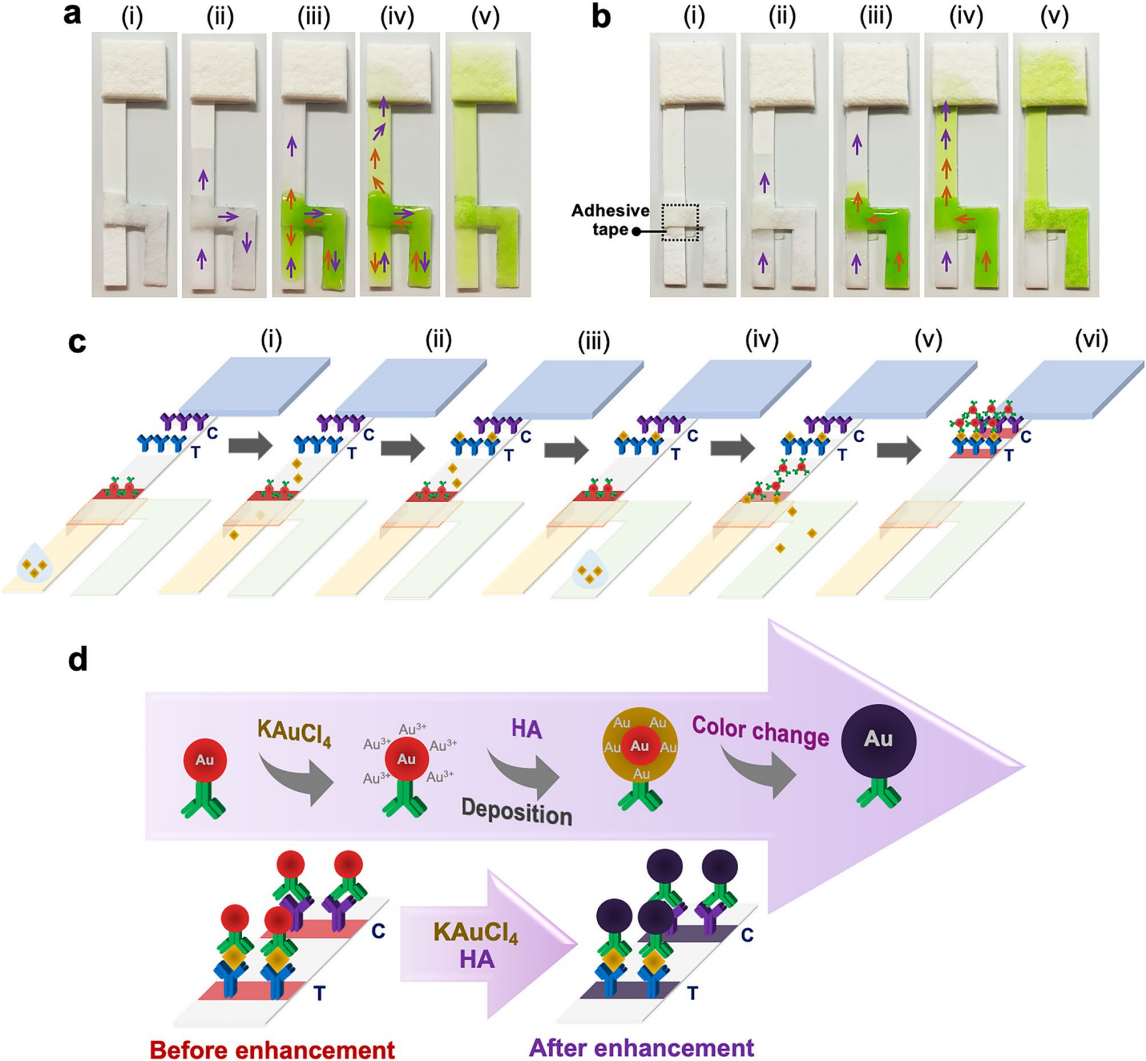
Solution flow on prototype devices (**a**) without and (**b**) with adhesive tape using milli-Q water and a food dye-colored solution as model liquids, with the flow of water (purple arrow) and green liquid (orange arrow) indicated: (1) prototype device before liquid deposition, (2) device after application of water on the first inlet pad, (3) device after application of green liquid on the second inlet pad, (4) device after the absorption of liquids into the absorbent pad, and (5) device after complete flow of solutions; (**c**) principle of the sensitivity improvement of ferritin colorimetric immunoassay using the double-sample inlet device: (1) sample loaded on the sample pad, (2) the target analyte flowing from the sample pad to the NCM, (3) the target analyte captured by the immobilized antibody, (4) sample loaded on the conjugate pad, (5) the free labeled antibody and the target analyte-labeled antibody complex flowing from the conjugate pad to the NCM and (6) the free labeled antibody captured by the target analyte bound to the immobilized antibody, and the target analyte-labeled antibody complex captured by the immobilized antibody, resulting in a red color on T and C lines; (**d**) schematic illustration of the enlargement of AuNPs after gold enhancement.

The key strategy of using an adhesive tape in the fabrication of the LFIA device was to separate two solutions independently flowing from two different inlet pads. Solution movements on devices without and with the adhesive tape were investigated by applying milli-Q water and green food dye-colored water on the first and second pad, respectively. By using the device without the adhesive tape (Fig. 1a), a controlled solution movement from each inlet towards the NCM alone could not be achieved after applying the water (purple arrow) and the green liquid (orange arrow) on the first (2) and second (3) inlet pads, respectively. Uncontrolled flow behavior including counter-flow occurred at the confluence point of the two liquids. After the absorption of all liquids by the absorbent pad (4–5), the flow of water and green liquid on the NCM appeared non-uniform. On devices incorporating the adhesive tape (Fig. 1b), the water (purple arrow) smoothly flowed from the first inlet pad toward the NCM, followed by the green liquid (orange arrow) from the second inlet pad also moving toward the NCM without interfering with the flow of the water (ii–v).

### Device working principle

The principle of the sensitivity improvement of AuNP-based LFIAs via the double-sample inlet device is shown Fig. 1c. Briefly, after loading the sample on the sample pad located under the adhesive tape (1), ferritin molecules present in the sample liquid flow to the NCM (2) and are captured by the immobilized antibody on the T line 3). After the sample is also loaded on the conjugate pad (4), ferritin molecules bind to some of the labeled antibodies located at the end of the conjugate pad, forming the target analyte-labeled antibody complex, while some free labeled antibody flows to the NCM (5). Finally, the free labeled antibody is captured by the ferritin bound immobilized antibody at the T line together with the target analyte-labeled antibody complex, while remaining free labeled antibodies are bound at the C line, leading to the appearance of red color bands on T and C lines (6). Based on the above procedure, there are two ways for the occurrence of binding interaction between the target analyte and the two antibodies, which might improve the sensitivity of the assay.

Furthermore, the principle of the sensitivity improvement of AuNP-based LFIAs using the gold enhancer (KAuCl_4_ and HA) is illustrated in Fig. 1d. Before gold enhancement, the target analyte, the labeled and the immobilized capture antibodies can bind into a sandwich form, resulting in creation of red color signals. After the flow of the gold enhancer solution^8^, AuNPs are enlarged by the reduction of Au^3+^ ions to bulk metal in the presence of HA as a reducing agent. In the reduction process, the Au^3+^ is rapidly deposited on the surface of the AuNP-labeled antibody, to form larger size AuNPs, resulting in the appearance of purple color signals.

### Parameter optimization

To achieve the best device efficiency with the use of suitable amounts of reagent and sample fluids as well as device materials for reducing operation costs, various parameters were systematically optimized: the concentration of anti–h ferritin 8803 for conjugation to AuNPs, the volume of the labeled antibody solution used for deposition onto conjugate pads, blocking conditions of the conjugate pad and the NCM, concentrations of anti–h ferritin 8806 and GAM antibody solutions applied on the NCM and finally, the concentrations of gold enhancer solutions^28^. The appropriate concentration of anti–h ferritin 8803 for conjugation to AuNPs during preparation of the labeled antibody was selected at 100 µg/mL, evaluated from the lowest concentration providing an unchanged solution color after adding 10% (w/v) NaCl and the highest red intensity (see Supplementary Fig. S1 online).

**Figure 2:**
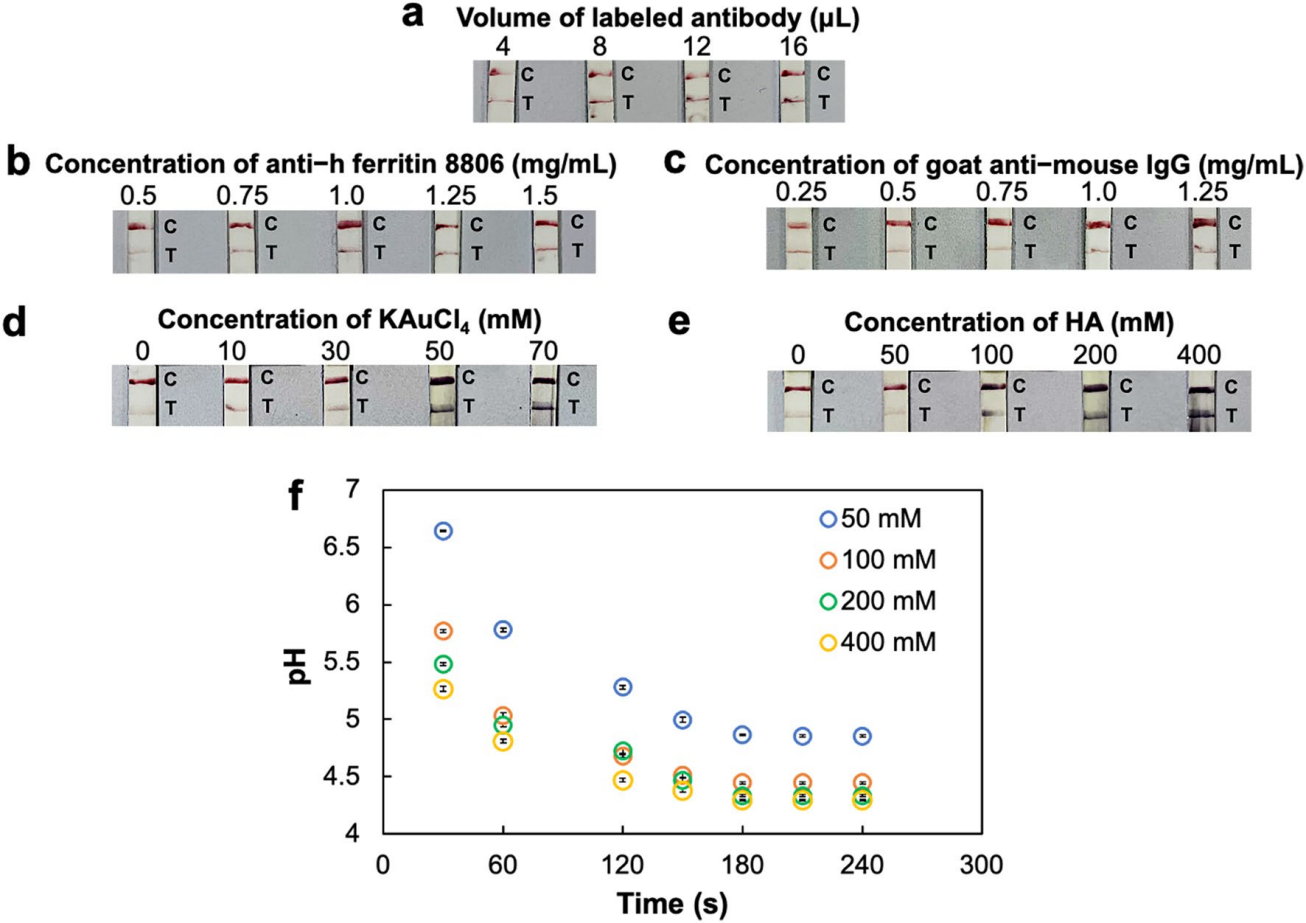
Optimization of parameters influencing the overall device performance: (**a**) effect of the volume of the labeled antibody deposited on conjugate pads (4–16 µL) on assays with 50 ng/mL ferritin samples; (**b**) effect of the concentration of anti-h ferritin 8806 (0.5–1.5 mg/mL) on the T line; (**c**) effect of the concentration of GAM (0.25–1.25 mg/mL) on the C line; (**d**) effect of the concentration of KAuCl_4_ (0–70 mM); (**e**) effect of the concentration of HA (0–400 mM) on assays with 20 ng/mL ferritin samples; (**f**) time-dependent solution pH decrease with HA/PVA/PEO blend films with various concentrations of HA (50–400 mM) soaked in water (n = 3).

In the case of colorimetric immunoassays, the amount of the two antibodies binding to the target analyte significantly affects the analytical efficiency^1^. There must be sufficient antibody molecules for target binding, in order to achieve a high signal intensity in colorimetric detection that can be clearly and easily evaluated via the naked eye. Various volumes of the above-mentioned optimized labeled antibody solution (4–16 µL) were evaluated by assaying 50 ng/mL of ferritin, a sufficient target concentration level for the simple and clear evaluation of color intensities at both T and C lines, using the double-sample inlet device. Results (Fig. 2a) indicated that 8 µL of the labeled antibody displayed a high visual color intensity equal to the 12 and 16 µL cases. Hence, to keep the device costs minimal, 8 µL were selected as the optimal volume for the labeled antibody applied on the device. In addition, for the improvement of labeled antibody release from the conjugate pad and movement on the NCM, the effects of the blocking solution on the conjugate pad and the NCM were investigated for three different conditions: (1) unblocked, (2) blocking with 3% (w/v) bovine serum albumin (BSA) and (3) blocking with 1 × blocking buffer. Applying 50 ng/mL of ferritin samples on the double-sample inlet devices (see Supplementary Fig. S2 online) with BSA-blocked conjugate pad (see Supplementary Fig. S2a online) and unblocked NCM (see Supplementary Fig. S2b online) enabled clear naked eye observation of both T and C lines and were selected as optimal blocking conditions.

Furthermore, concentrations of the immobilized capture antibody (0.5–1.5 mg/mL) and the GAM (0.25–1.25 mg/mL) applied to fabricate the T and C lines were studied to select the smallest concentrations exhibiting good analytical performance. These investigations were performed by applying 20 ng/mL of ferritin samples (an average cut-off level of ferritin in humans) on the double-sample inlet device. The results indicated that 1.0 mg/mL of the immobilized capture antibody (Fig. 2b) and 0.5 mg/mL of GAM (Fig. 2c) are the minimal concentrations showing appropriate color intensities and were therefore chosen as optimal values for the preparation of the T and C lines on the NCM.

To fabricate the gold enhancement device, the concentrations of KAuCl_4_ (0–70 mM) and HA (0–400 mM) were investigated in assays performed with 20 ng/mL of ferritin samples. Accordingly, 50 mM KAuCl_4_ (Fig. 2d) and 200 mM HA (Fig. 2e), which were the minimal concentrations exhibiting appropriate color intensity enhancement with low background color interference, were selected as optimal values for gold enhancement. Generally, KAuCl_4_ and HA had to be separately applied on the device at different positions, because their mixed solutions immediately turned to purple color. For this reason, the HA/PVA/PEO blend film was utilized for applying HA in this study in contrast to directly depositing and drying of HA on the gold pad (see Supplementary Fig. S2c online). Results showed that the use of the HA/PVA/PEO blend film provided a color signal improvement, whereas, by directly depositing HA, KAuCl_4_ and HA interacted and adsorbed on the gold pad, resulting in purple color development on the gold pad instead of the enhancement of the color intensity at T and C lines on the NCM. For further investigation, the HA/PVA/PEO blend films were soaked in water, and the pH of the solution was measured every 30 s as an indicator of HA release from the blend films compared to the pH of the 200 mM HA solution (pH = 3.01). As the results in Fig. 2f demonstrate, the solution pH decreased with increasing film soaking times from 0 to 180 s and with increasing HA concentrations used for film preparation, confirming the gradual release of HA from the HA/PVA/PEO blend films. Moreover, the pH of the solution was stable after 180 s with a pH value of 4.34 measured for films prepared from the optimal concentration of 200 mM HA. From these results, it could be confirmed that the use of the HA/PVA/PEO blend film enables a delayed release (180 s) and flow of HA on the device. Therefore, after application of water on gold pads modified with polymer blend films, KAuCl_4_ reaches the T and C lines to react with AuNPs before the release of HA over 180 s, which could significantly improve the assay sensitivity. All optimal parameters influencing the overall device performance are summarized in Supplementary Table S1 online.

### Characterization of HA/PVA/PEO blend film and gold enlargement

**Figure 3:**
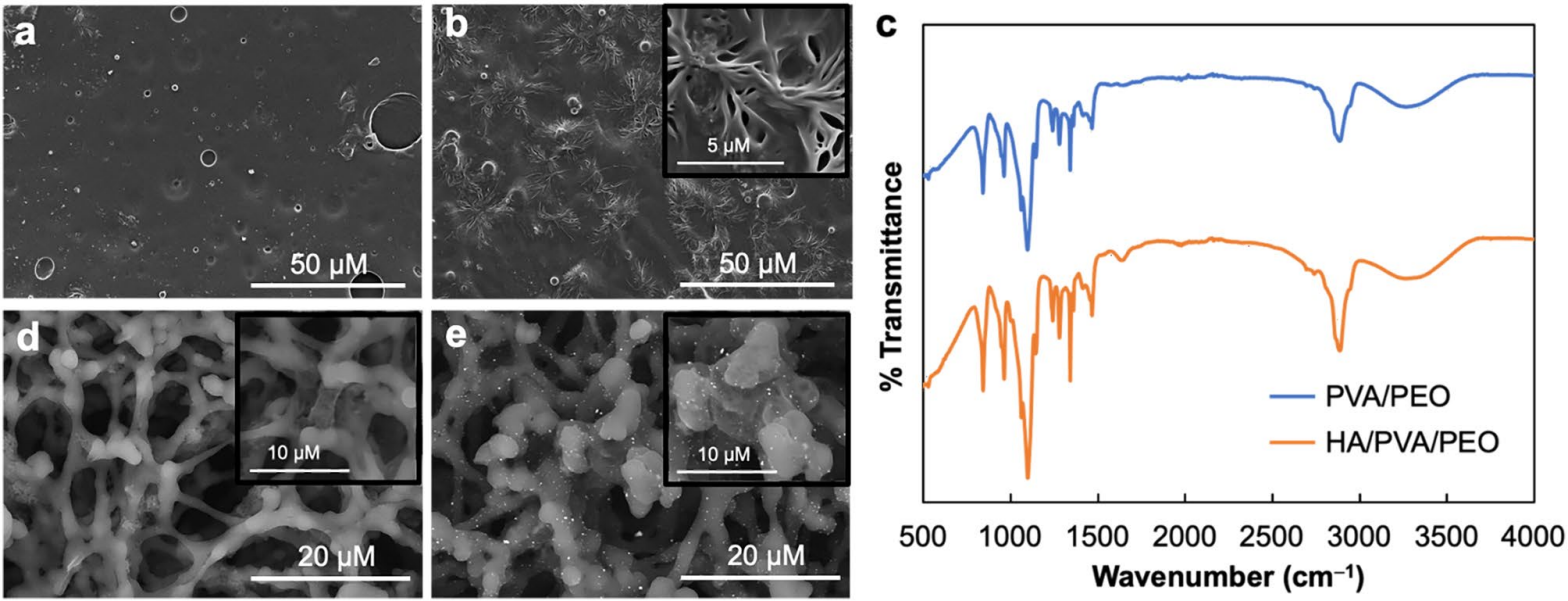
SEM images of (**a**) PVA/PEO and (**b**) HA/PVA/PEO blend films with magnifications of 1000× and 10,000× (inset); (**c**) FTIR spectra of PVA/PEO and HA/PVA/PEO blend films; SEM images of T lines on the NCM (**d**) before and (**e**) after gold enlargement with magnifications of 2500× and 5000× (inset) using backscattered electron (BSE) mode.

To confirm the successful preparation of the polymer blend film, the surface morphology of the HA/PVA/PEO blend film was characterized by SEM and compared to that of a PVA/PEO blend film. The SEM image of the PVA/PEO blend film displayed a smooth surface (Fig. 3a). After the addition of HA (Fig. 3b), a 3D fractal structure was found within the PVA/PEO blend film, confirming the successful immobilization of HA^29^. Furthermore, FTIR of the HA/PVA/PEO blend film was also investigated and compared to that of the PVA/PEO blend film (Fig. 3c). There is a strong broad band in the 3200–3400 cm^−1^ region attributed to O–H and N–H vibrations of the COOH and HNOH groups of the HA/PVA/PEO blend film, respectively. The band at approximately 2885 cm^−1^ can be assigned to C–H bonds in the PVA/PEO polymer structure. The small band at around 1241 cm^–1^ is assigned to CH_2_ symmetric twisting. Strong bands observed at 1086 cm^−1^ were recognized as crystallization sensitive bands, referring to the C–O stretching vibration. A sharp band at 840 cm^–1^ is attributed to C–O stretching^30–32^. The characteristic peak for amino groups of HA is at approximately 1636 cm^−1^, confirming the achievement of the immobilization of HA within the PVA/PEO blend film^33^.

To characterize the gold enlargement complex by the reduction of Au^3+^ ions to bulk metal in the presence of HA on the device, the surface morphologies of T lines on the NCM before (the initial AuNPs) and after gold enlargement (gold enhanced AuNPs) were investigated using SEM. The SEM images indicated that the size of AuNPs (Fig. 3e) significantly increased after gold enlargement compared to the initial AuNPs before the enlargement (Fig. 3d). The gold enlargement complex with the average size of 3.09 ± 0.43 µm (n = 20) exhibited a narrow size distribution and uniform particles size^34,35^, assuming the high reproducibility of the reaction.

### Selectivity and storage stability

**Figure 4:**
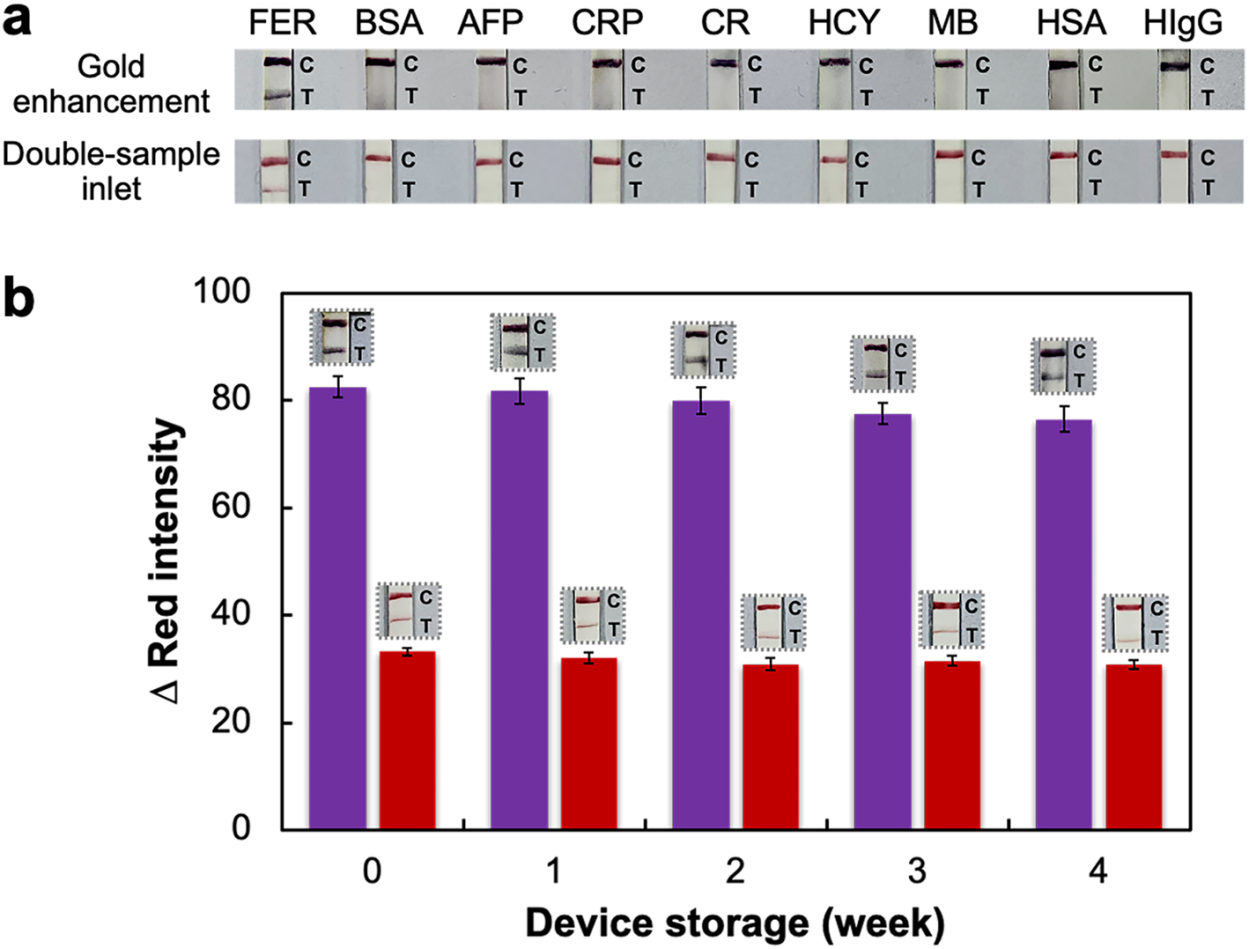
(**a**) Selectivity of the gold enhancement and double-sample inlet devices after the application of 20 ng/mL of ferritin (FER), 10% (w/v) BSA, 1 µg/mL of alpha-fetoprotein (AFP), c-reactive protein (CRP), creatinine (CR), homocysteine (HCY), myoglobin (MB), human serum albumin (HSA) and human immunoglobulin G (HIgG); (**b**) storage stability of gold enhancement (purple bars) and double-sample inlet (red bars) devices after keeping at RT in a desiccator between 0 and 4 weeks and measuring 20 ng/mL of ferritin (n = 3).

The selectivity of the proposed devices was investigated by comparing the results obtained with different potential interferents existing in human serum and blood^36^, including 10% (w/v) BSA, 1 µg/mL of alpha-fetoprotein (AFP), c-reactive protein (CRP), creatinine (CR), homocysteine (HCY), myoglobin (MB), human serum albumin (HSA) and human immunoglobulin G (HIgG), with those of 20 ng/mL of ferritin. Results in Fig. 4a indicated that there were no measurable color intensities on the T line after applying solutions of the interferents, verifying a high selectivity toward ferritin due to the highly specific binding interaction between two monoclonal antibodies and the antigen.

In addition, the storage stability of the gold enhancement and double-sample inlet devices was also investigated after keeping these devices at RT in a desiccator for 0–4 weeks and testing with 20 ng/mL of ferritin. As illustrated in Fig. 4b, the observed changes in the Δred intensity value for 20 ng/mL of ferritin remained above 93% of their initial intensities, verifying the stability of the proposed devices.

### Analytical performance

**Figure 5:**
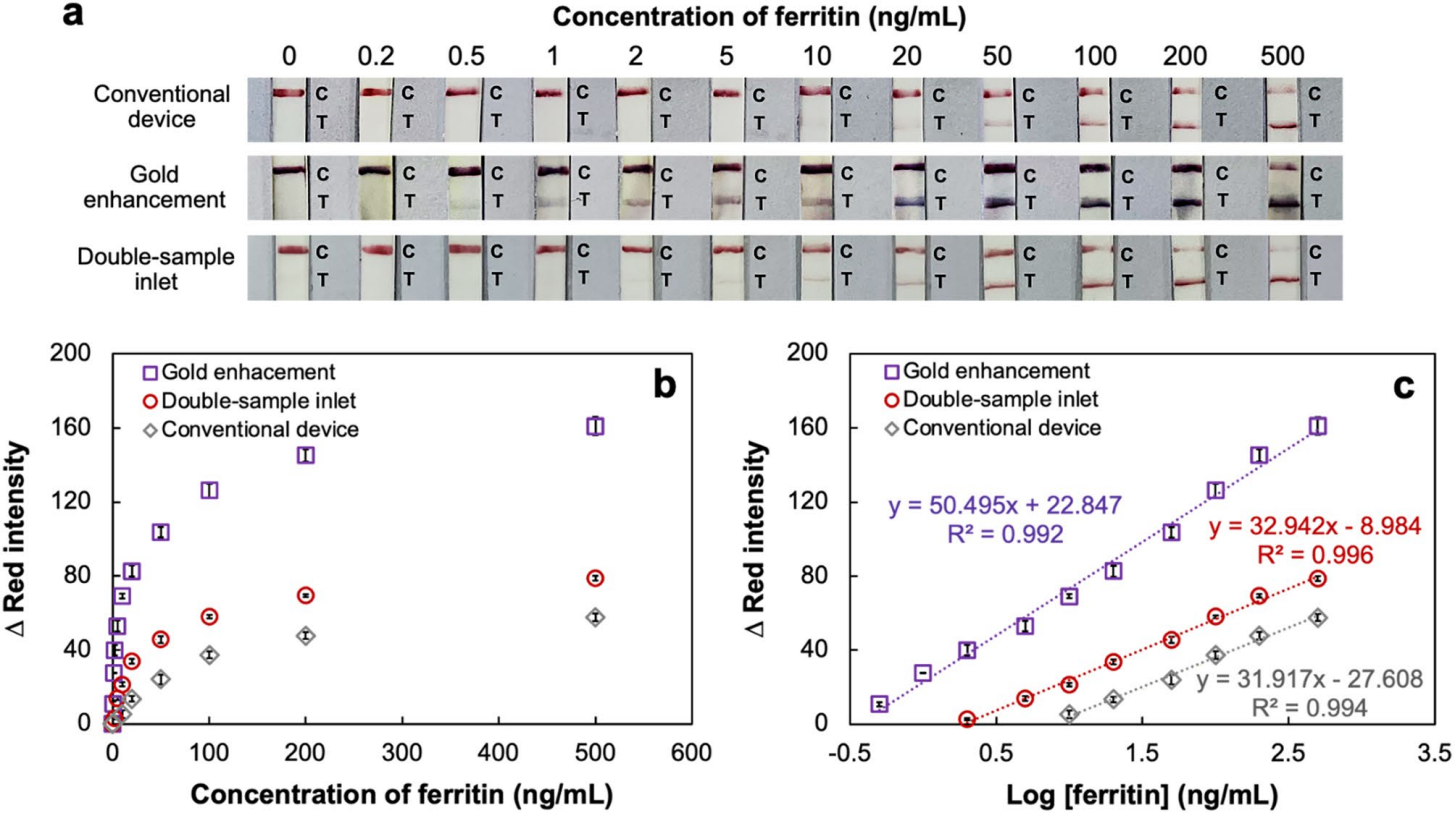
(**a**) Photographs after applying ferritin solutions between 0 and 500 ng/mL on conventional, gold enhancement and double-sample inlet devices; (**b**) the relationship between the Δred intensity and the concentration of ferritin examined in triplicate (n = 3); (**c**) the linearities of the Δred intensity versus the logarithm of the ferritin concentration (n = 3).

The proposed devices were utilized for the measurement of various concentrations (0–500 ng/mL) of ferritin, and the overall assays were completed within 10 min without any requirement of external equipment or instruments. The increase of the color intensity that appeared on the T line correlated with the increase of the ferritin concentration, and the visual limit of detection (LOD) was determined as the minimal concentration of ferritin resulting in naked eye observable color intensity on the T line. From the photographs in Fig. 5a, visual LODs achieved with the conventional (see experimental procedure in Supplementary S1 and Fig. S3 online), gold enhancement and double-sample inlet devices were respectively found to be 10, 0.5 and 2 ng/mL, demonstrating that the gold enhancement and double-sample inlet deS3vices provided 20-fold and fivefold greater sensitivities than the conventional device, respectively. In terms of quantitative analysis (Fig. 5b), ferritin concentration-dependent Δred intensities were examined in triplicate, indicating that the Δred intensities obtained with the gold enhancement and double-sample inlet devices were evidently higher than those of the conventional device. Quantitative calibration curves of the Δred intensity as a function of the logarithm of the ferritin concentration shown in Fig. 5c displayed linearities with good correlations for the gold enhancement device (0.5–500 ng/mL, y = 50.495x + 22.847 R^2^ = 0.992) and the double-sample inlet device (2–500 ng/mL, y = 32.942x – 8.984, R^2^ = 0.996), while the small error bars representing the standard deviations (n = 3) indicated high reproducibility. After reaction completion, the results were founded that there was some solution residue in the conjugate pad area. However, in the calibration curves, small error bars were observed with an acceptable relative standard deviation (RSD) below 3.9%. Therefore, this solution residue did not affect the accuracy of the detection. The calculated LODs of the gold enhancement and double-sample inlet devices obtained from the calibration curves were respectively found to be 0.03 and 0.05 ng/mL (LOD = 3SD/slope)^8^. Compared to other previous reports of ferritin LFIAs as summarized in Supplementary Table S2 online, the proposed device provided improved detection limits and a satisfactory concentration response range.

Furthermore, the proposed device was also compared several previous reports on signal amplification of AuNP-based LFIAs using a gold enhancer as shown in Table 1. The signal amplification achieved in these previous reports was 5–100 folds after gold enhancement, and the overall reaction was complete within 15–20 min, showing that our proposed device gave a shorter analysis time with a high signal amplification value. To achieve 8–100 folds signal amplification^9,34,37,38^, the procedure applied in these previous reports required multiple steps of sample loading until the reaction was complete, gold enhancer solution application and washing. In addition, the enhancement process required the use of freshly prepared gold enhancer solutions, leading to more demanding assays and short-term stability. Panraksa et al.^8^ also reported a one-step gold-enhanced AuNP-based LFIA by fabricating a wax-printed patterned LFIA platform that required an expensive wax printer and provided a minimal signal amplification value (fivefold). Furthermore, no device storage study was conducted because of presumably short-term stability. Therefore, the proposed gold enhancement device integrating the adhesive tape and the HA/PVA/ PEO blend film exhibited numerous benefits, including rapid analysis (10 min), long-term stability (at least 1 month) and simple fabrication and use, which could be suitable and acceptable for on-site measurements.

**Table 1:** Comparison of the signal amplification of AuNP-based LFIAs using a gold enhancer.

Analyte	Reactant	Enhancement system	LOD	Linear range	Signal amplification (folds)	Analysis time (min)	References
Avian influenza and Newcastle disease virus	1% (w/v) HAuCl_4_/10 mM HA	Washing and soaking in freshly prepared enhancer	2^−12^	2^1^–2^−9^	100	15	^37^
*Escherichia coli* O157:H7	1% (w/v) HAuCl_4_/ 10 mM HA	Washing and adding freshly prepared enhancer	5 × 10^3^ CFU/mL	–	8	20	^38^
*Salmonella* Enteritidis	1% (w/v) HAuCl_4_/10 mM HA	Adding freshly prepared enhancer to sample pad	10^4^ CFU/mL	10^3^–10^8^ CFU/mL	100	20	^9^
*Ralstonia solanacearum*	1% (w/v) HAuCl_4_/2 mM HA	Applying freshly prepared enhancer on NCM	3 × 10^4^ cells/mL	–	33	15	^34^
C-reactive protein	200 mM KAuCl_4_/750 mM HA	Single step operation by wax-printed sequential flow device	0.001 µg/mL	0.1–5 µg/mL	5	15	^8^
Ferritin	50 mM KAuCl_4_/200 mM HA	Double inlet device based on adhesive tape and polymer blend film	0.03 ng/mL	0.5–500 ng/mL	20	10	This work

### Real sample application

The practical applicability was tested by directly applying human serum samples on the proposed devices without any pretreatment or dilution. Results in Table 2 reveal that the concentration levels of ferritin in the three sera measured with the proposed gold enhancement device are not significantly different from those obtained by the enzyme-linked immunosorbent assay (ELISA) standard method (see experimental procedure in Supplementary S2 online). Test values from three-independent measurements were found to be 98–101% accurate with relative standard deviation (RSD) below 2.5%. These results verified that the proposed device provided high accuracy and could be utilized for early screening and measuring the amount of ferritin in biological samples.

**Table 2:** Results for using the proposed device for colorimetric immunoassay of ferritin in human serum samples (n = 3).

Sample	Ferritin level (ng/mL)		Accuracy (%)	RSD (%)
	Proposed device	ELISA		
Serum 1	16.14 ± 0.2	16.53	98	1.3
Serum 2	62.59 ± 0.9	63.87	98	1.5
Serum 3	23.37 ± 0.6	23.13	101	2.5

## Conclusions

A novel device based on a low-cost adhesive tape and a HA/PVA/PEO blend film was firstly proposed for the sensitivity improvement of AuNP-based LFIAs. The adhesive tape facilitated the separation of two solutions independently flowing from the different inlet pads, and the HA/PVA/PEO blend film could slow down the release and flow of the HA reducing agent entrapped in the polymer blend film for 180 s. The improvement of the assay sensitivity was achieved by using an independent double-sample inlet device and a gold enhancement device via the catalytic reaction of KAuCl_4_ and HA. Using the devices under optimal conditions with overall reaction times of 10 min, they provided lower limits of detection than the traditional AuNP-based device with high selectivity and long-term stability. In addition, the application of the device to determine ferritin in human serum samples exhibited high accuracy. Consequently, the designed device could be further applied for sensitivity improvement requiring an additional procedure (e.g. signal amplification using other metal enhancer solutions and enhancement of colorimetric signal using an enzyme-labeling cooperated with a substrate).

## Materials and methods

### Chemicals and materials

All chemicals and materials used in this work are presented as Supplementary S2 online.

### Preparation of the labeled antibody

The procedure of the anti-h ferritin antibody 8803 conjugation to AuNPs is presented as Supplementary S4 online.

### Preparation of the HA/PVA/PEO blend film

Based on previous reports, a solid polymer blend film containing the reducing agent was formed using PVA and PEO as carrier polymers via the solution-casting technique with a slight modification^39,40^. First, 12% (w/v) PVA, 3% (w/v) PEO and various concentrations (0–400 mM) of HA as reducing agent were added into water. The prepared solution was then heated at 120 °C under continuous stirring at 600 rpm until homogeneity was obtained. Next, the solution was casted using a cylindrical film applicator with a width of 100 mm and a gap of 200 µM (VF 1523; TQCSHEEN BRAND, Netherland), and was then left to dry until the film was formed. Finally, the obtained HA/PVA/PEO blend film was punched with a diameter size of 4 mm and kept in a desiccator for further use. The prepared HA/PVA/PEO blend film was characterized by scanning electron microscopy (SEM) (JSM-6400; Japan Electron Optics Laboratory Co., Ltd., Japan) and Fourier transform infrared spectroscopy (FTIR) (PerkinElmer, Waltham, MA, USA).

### LFIA device fabrication

**Figure 6:**
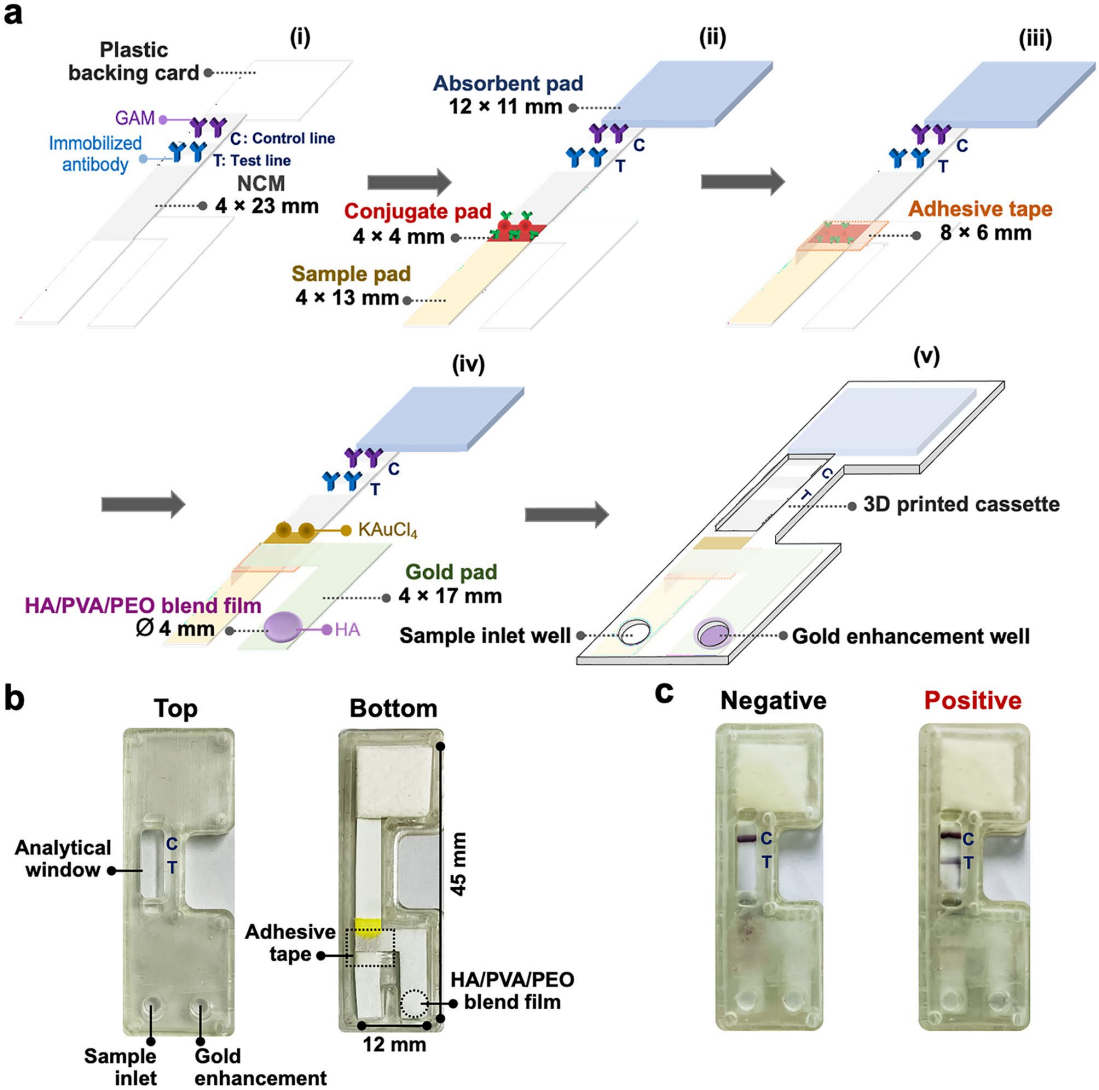
(**a**) Fabrication procedure of the gold enhancement device: (1) preparation and attachment of the NCM on the plastic backing card, (2) preparation and attachment of the conjugate, absorbent and sample pads, (3) attachment of the adhesive tape, (4) preparation and attachment of the gold pad and the HA/PVA/PEO blend film and (5) placement of the device into the 3D printed cassette; (**b**) top and bottom views of the gold enhancement device (12 mm wide and 45 mm long) inside the 3D printed cassette comprising a sample inlet well, a gold enhancement well and an analytical window labeled with letters T and C; (**c**) representative data of negative and positive (20 ng/mL of ferritin) results after gold enhancement.

Devices for the sensitivity improvement of AuNP-based LFIAs were designed on two platforms: (1) a gold enhancement device based on a low-cost adhesive tape and an HA/PVA/PEO blend film and (2) an independent double-sample inlet device based on the adhesive tape. The gold enhancement device was fabricated as illustrated in Fig. 6a. First, the NCM (4 × 23 mm) was prepared by stamping 0.4 µL of anti-h ferritin 8806 solution as an immobilized capture antibody and 0.4 µL of goat anti-mouse IgG (GAM) solution using a glass slide (fixed area: 4 × 1 mm) to create the test (T) and control (C) lines, respectively, with a gap of 4 mm between them, followed by drying at room temperature (RT) in a desiccator for 1 h and attachment to a plastic backing card (1). After that, 8 µL of the labeled antibody was applied on the conjugate pad (4 × 4 mm), followed by drying at RT in the desiccator for 1 h. The prepared conjugate pad and the absorbent pad (12 × 11 mm) were then attached to the backing card overlapping different sides of the NCM by 2 mm, followed by the attachment of the sample pad (4 × 13 mm) overlapping the conjugate pad by 2 mm (2). After that, the low-cost adhesive tape (8 × 6 mm) was placed on a position totally covering the conjugate pad, overlapping the sample pad for 2 mm and the NCM for 4 mm (3). 8 µL of KAuCl_4_ solution was applied on the top of the gold pad (4 × 17 mm with a special pattern), followed by drying at RT in the desiccator for 1 h and attached on the backing card and the adhesive tape, overlapping the NCM by 2 mm. The 4-mm diameter HA/PVA/PEO blend film was then placed at the bottom of the gold pad (4). Finally, the prepared device was inserted into a 3D-printed cassette (5), consisting of a sample inlet well, a gold enhancement well and an analytical window labeled with the letters T and C. The top and bottom views of the 12-mm wide and 45-mm long device inserted in the 3D printed cassette are shown in Fig. 6b. The independent double-sample inlet device was prepared according to the fabrication procedure of the gold enhancement device with a slight difference as Supplementary S5 online.

### Assay performance evaluation

To evaluate the assay performance, ferritin samples (0–500 ng/mL in 0.01 M phosphate buffered saline (PBS), pH 7.4) as the model analyte were applied on the gold enhancement and the double-sample inlet devices. To measure the sample on the double-sample inlet device, 100 µL of the sample was first dropped into the sample inlet well, and after a waiting time of 2 min another 100 µL of sample was loaded into the well of the conjugate pad. The solutions were allowed to flow until the reaction was complete, and the resulting data were visually evaluated in the analytical window. As Supplementary Fig. S4c online, in the presence of ferritin (positive), ferritin specifically bound to the immobilized and the labeled antibodies in a sandwich form, producing red color signals on both T and C lines. On the contrary, zero or an unmeasurably low amount of ferritin (negative) resulted in the appearance of only the C line signal. In the case of the gold enhancement device, the measurement was performed by firstly dropping 100 µL of sample into the sample inlet well, followed by loading 100 µL of water into the gold enhancement well 2 min after sample application. After complete reaction, the positive result displayed purple color signals on both T and C lines, whereas the negative result exhibited only one C line (Fig. 6c). Furthermore, quantitative measurement was also performed using a smartphone for picture capture and ImageJ for data analysis, whereby the area of the T line selected for color analysis was fixed.

### Ethics statement

The human serum samples were obtained from healthy volunteers approved by the Faculty of Medicine, Chulalongkorn University, Thailand, and all procedures performed in studies involving human participants were in accordance with the ethical standards of the institutional research committee from Chulalongkorn Hospital, Bangkok, Thailand. Informed consent was obtained from all healthy volunteers prior to sample collection.

## Supplementary Information

Supplementary Information.

## Data Availability

The datasets generated during and/or analyzed during the current study are available from the corresponding author on reasonable request.
