# Supplementary material for: Improvement in sensitivity for lateral flow immunoassay of ferritin using novel device design based on gold-enhanced gold nanoparticles
Source: Sci Rep. 2022 May 12;12:7831. doi: 10.1038/s41598-022-11732-5 (PMC9098456; doi:10.1038/s41598-022-11732-5)
Supplement: Supplementary file 1 — Supplementary Information. [file 41598_2022_11732_MOESM1_ESM.docx]

**Supplementary information**

**Improvement in sensitivity for lateral flow immunoassay of ferritin using novel device design based on gold-enhanced gold nanoparticles**

Pattarachaya Preechakasedkit^1^, Kanyapat Teekayupak^2^, Daniel Citterio^3^, Nipapan Ruecha^1, 2*^

^1^ Metallurgy and Materials Science Research Institute, Chulalongkorn University, Soi Chula 12, Phayathai Rd., Pathumwan, Bangkok 10330, Thailand

^2^ Electrochemistry and optical spectroscopy Center of Excellence (EOSCE), Department of Chemistry, Faculty of Science, Chulalongkorn University, Pathumwan, Bangkok 10330, Thailand

^3^ Department of Applied Chemistry, Faculty of Science and Technology, Keio University, Yokohama, Kanagawa 223-8522, Japan

^*^ Corresponding author Email: nipapan.r@chula.ac.th


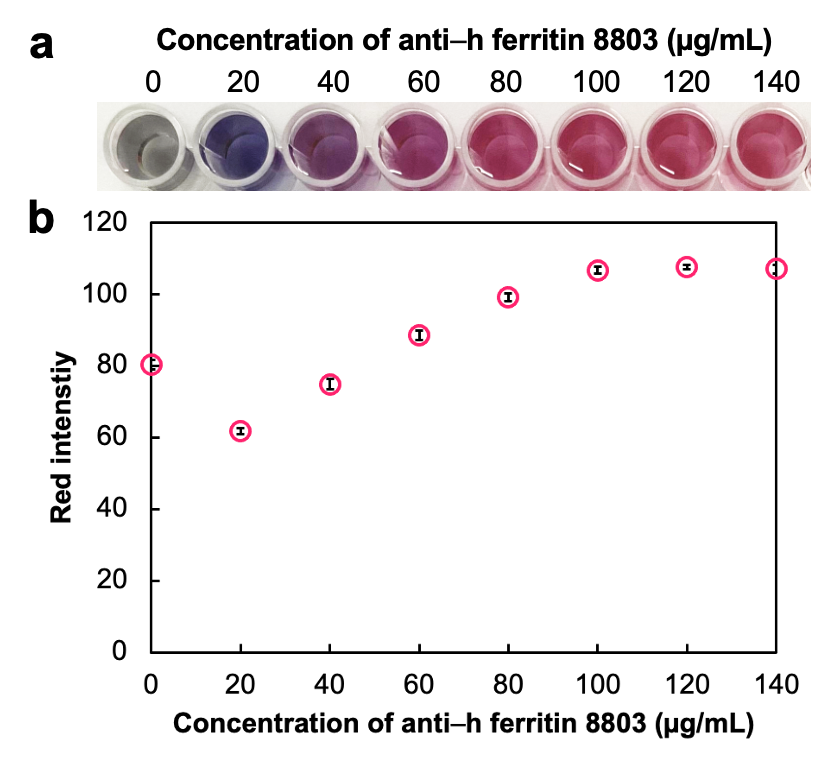


**Figure S1** Optimization of the anti–h ferritin antibody 8803 concentration ranging from 0–140 µg/mL for conjugation to AuNPs: a) photograph of the color change after adding 10% (w/v) NaCl and b) relationship between the red intensity and the concentration of anti–h ferritin antibody 8803 (n = 3)

**
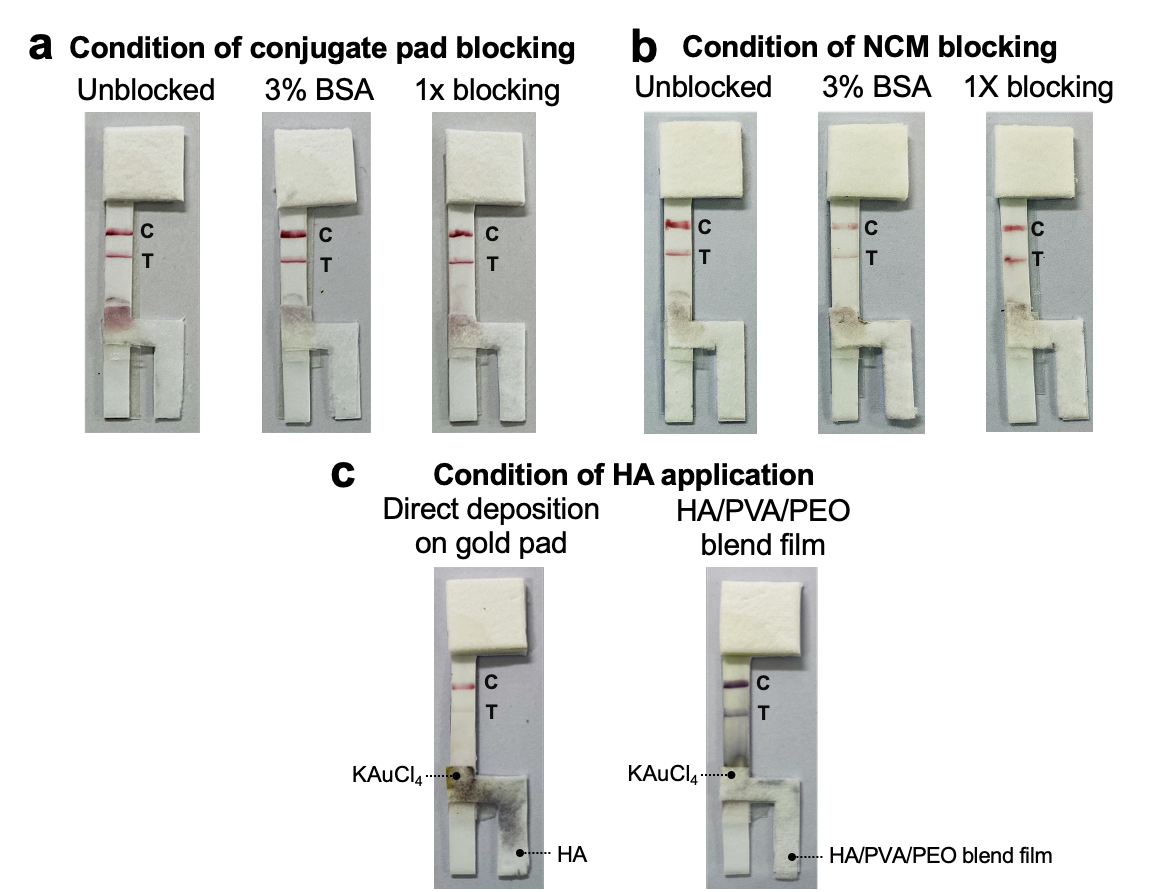
**

**Figure S2** Effect of the blocking solution on a) the conjugate pad and b) the NCM using 3% (w/v) BSA and 1x blocking buffer as the blocking solution compared to the absence of any blocking after applying 50 ng/mL of ferritin; c) effect of HA application through direct deposition on the gold pad and the HA/PVA/PEO blend film after applying 20 ng/mL of ferritin

**Table S1** Summary of optimal parameters influencing the overall device performance

| **Parameter** | **Range of variation** | **Criteria for selection** | **Optimal value** |
| --- | --- | --- | --- |
| Concentration of anti–h ferritin 8803 for conjugation to AuNPs | 0–140 µg/mL | The lowest concentration providing an unchanged solution color after adding 10% (w/v) NaCl and the highest red intensity | 100 µg/mL |
| Volume of the labeled antibody solution | 4–16 µL | The minimal volume providing a high visual color intensity | 8 µL |
| Blocking solution on the conjugate pad | unblocked, blocking with 3% (w/v) BSA, blocking with 1x blocking buffer | The condition showing clear naked eye observation of both T and C lines | BSA-blocked conjugate pad |
| Blocking solution on the NCM | unblocked, blocking with 3% (w/v) BSA, blocking with 1x blocking buffer | The condition showing clear naked eye observation of both T and C lines | Unblocked NCM |
| Concentration of anti–h ferritin 8806 (T line) | 0.5–1.5 mg/mL | The minimal concentration showing appropriate color intensity | 1.0 mg/mL |
| Concentration of GAM (C line) | 0.25–1.25 mg/mL | The minimal concentration showing appropriate color intensity | 0.5 mg/mL |
| Concentration of KAuCl_4_ | 0–70 mM | The minimal concentrations exhibiting appropriate color intensity enhancement with low background color interference | 50 mM |
| Concentration of HA | 0–400 mM | The minimal concentrations exhibiting appropriate color intensity enhancement with low background color interference | 200 mM |

**S1. Conventional LFIA device**

The conventional LFIA device was prepared by first stamping 0.4 µL of the capture antibody solution and 0.4 µL of GAM solution on the NCM (4 × 23 mm) using a glass slide to create the T and C lines, respectively, with a gap of 4 mm between them, followed by drying at RT in a desiccator for 1 h and attachment to a plastic backing card. Then, 8 µL of the labeled antibody was applied on the conjugate pad (4 × 4 mm), followed by drying at RT in the desiccator for 1 hour. The prepared conjugate pad and the absorbent pad (4 × 11 mm) were then attached to the backing card overlapping different sides of the NCM by 2 mm, followed by the attachment of the sample pad (4 × 13 mm) overlapping the conjugate pad by 2 mm. The 4-mm wide and 45-mm long device was used for measurements by dropping 100 µL ferritin samples on the sample pad and allowing to flow until the complete reaction (10 min).


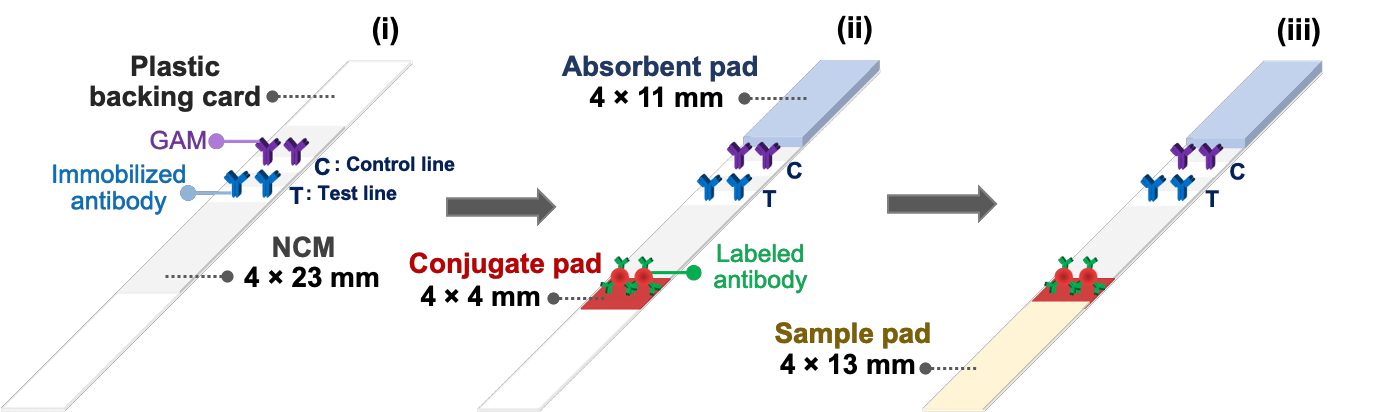


**Figure S3** Fabrication procedure of the conventional LFIA device: (i) preparation and attachment of the NCM on the plastic backing card, (ii) preparation and attachment of the conjugate and absorbent pads and (iii) attachment of the sample pad

**Table S2** Comparison among various LFIAs of ferritin

| **Labeling material** | **Linearity (ng/mL)** | **LOD (ng/mL)** | **Ref.** |
| --- | --- | --- | --- |
| Gold nanoparticles | 10–20,000 | 10 | ^1^ |
| Carbon nanotubes | 100–5,000 | 50 | ^2^ |
| Gold nanoparticles | 3–556 | 11 | ^3^ |
| Rhodium nanoparticles | 1–500 | 0.3 | ^4^ |
| Gold-enhanced gold nanoparticles | 0.5–500 | 0.03 | This work |

**S2. Validation method by ELISA**

The measurement of the ferritin level in the human serum sample using the proposed device was validated with the ELISA standard method according to the following procedure. First, 100 µL of 1 µg/mL of the capture antibody solution was coated on a 96-microwell plate at 4°C overnight, followed by a washing step. Then, 300 µL of 5% (w/v) skim milk was added and incubated at 37°C for 1 h to block the unbound surface, followed by a washing step. After that, 100 µL of various concentrations (0–200 ng/mL) of ferritin and serum sample were added and incubated at 37°C for 1.5 h, followed by a washing step. Next, 100 µL of 1:1,000 HRP labeled antibody was added and incubated at 37°C for 2 h. After the washing step, 100 µL of 3,3′,5,5′-tetramethylbenzidine (TMB) containing hydrogen peroxide (H_2_O_2_) was added and kept in the dark for 15 min. Finally, 100 µL of 1 M sulfuric acid (H_2_SO_4_) was added to stop the reaction, followed by measuring the absorbance at 450 nm.

**S3. Chemicals and materials**

Alpha–fetoprotein (AFP), bovine serum albumin (BSA), creatinine, c-reactive protein (CRP), gold nanoparticles (AuNPs, ~20 nm particle size), homocysteine, hydroxylamine hydrochloride (HA; NH_2_OH•HCl), myoglobin, phosphate buffered saline (PBS) tablets, polyethylene oxide (PEO, Mw. 100,000), polyvinyl alcohol (PVA, Mw. 89,000-98,000), sodium chloride (NaCl), goat anti–mouse IgG (GAM), human serum albumin (HSA) and human immunoglobulin G (HIgG) were purchased from Sigma Aldrich (St. Louis, MO, USA). Potassium tetrachloroaurate (III) *n*-Hydrate (KAuCl_4_) was purchased from Wako Pure Chemical Industries Ltd (Osaka, Japan). Block PRO^TM^ protein-free blocking buffer (20x) was purchased from Energenesis Biomedical Co., Ltd. (Taipei, Taiwan). Natural human ferritin protein (ab96514) was obtained from Abcam (Cambridge, United Kingdom). Anti–h ferritin monoclonal antibodies (8806 as an immobilized capture antibody and 8803 as a labeled antibody) were purchased from MedixBiochemica (Espoo, Finland). All buffers and solutions were prepared using 18Ω milli-Q water. The low-cost adhesive tape (Scotch® packaging tape) was obtained from 3M Thailand Co., Ltd (Bangkok, Thailand). The glass fiber membrane (Standard 17) used as conjugate and gold pads, nitrocellulose membrane (AE100), absorbent pad (CF7) and sample pad (Fusion 5) were purchased from Whatman-GE Healthcare (Pittburgh, PA, USA).

**S4. Preparation of the labeled antibody**

The anti–h ferritin antibody 8803 was conjugated to the AuNPs to obtain the labeled antibody. At first, the concentration of the anti–h ferritin antibody 8803 was optimized ^5^. Various concentrations (0–140 µg/mL) of the antibody were briefly mixed with AuNPs (pH 8.0, 20 nm particle size) for 30 min, followed by the addition of 10% (w/v) NaCl. The optimal concentration was selected by visually evaluating the color change and analyzing the red intensity via the ImageJ software (NIH, Bethesda, MD, USA). After obtaining the appropriate concentration, the labeled antibody was prepared by mixing the AuNPs with an appropriate concentration of the anti–h ferritin antibody 8803 and incubation for 1 h at room temperature (RT). Then, nonspecific sites were blocked by directly adding 1x blocking buffer at RT for 1 h. Finally, the mixture was centrifuged at 15,000 rpm for 30 min, followed by removing the supernatant and resuspending in 1x blocking buffer. Finally, the obtained labeled antibody was stored at 4°C.

**S5. Fabrication of the independent double-sample inlet device**

The independent double-sample inlet device was prepared according to the fabrication procedure of the gold enhancement device with a slight difference (Fig. S4a). After the attachment of the prepared NCM on the backing card (i), the sample (4 × 15 mm) and the absorbent pads were attached to the backing card overlapping different sides of the NCM by 2 mm without any pretreatment (ii). This was followed by the attachment of the adhesive tape covering the sample pad and the NCM for 2 and 4 mm, respectively (iii). Then, 8 µL of the labeled antibody was applied and dried on the top of the conjugate pad (4 × 17 mm with a special pattern), followed by the attachment on the backing card and the adhesive tape, overlapping the NCM by 2 mm (iv). Finally, the prepared device was also inserted into the 3D printed cassette (v), consisting of double-sample inlet wells and the analytical window. The top and bottom views of the device inserted in the 3D printed cassette are shown in Fig. S4b.


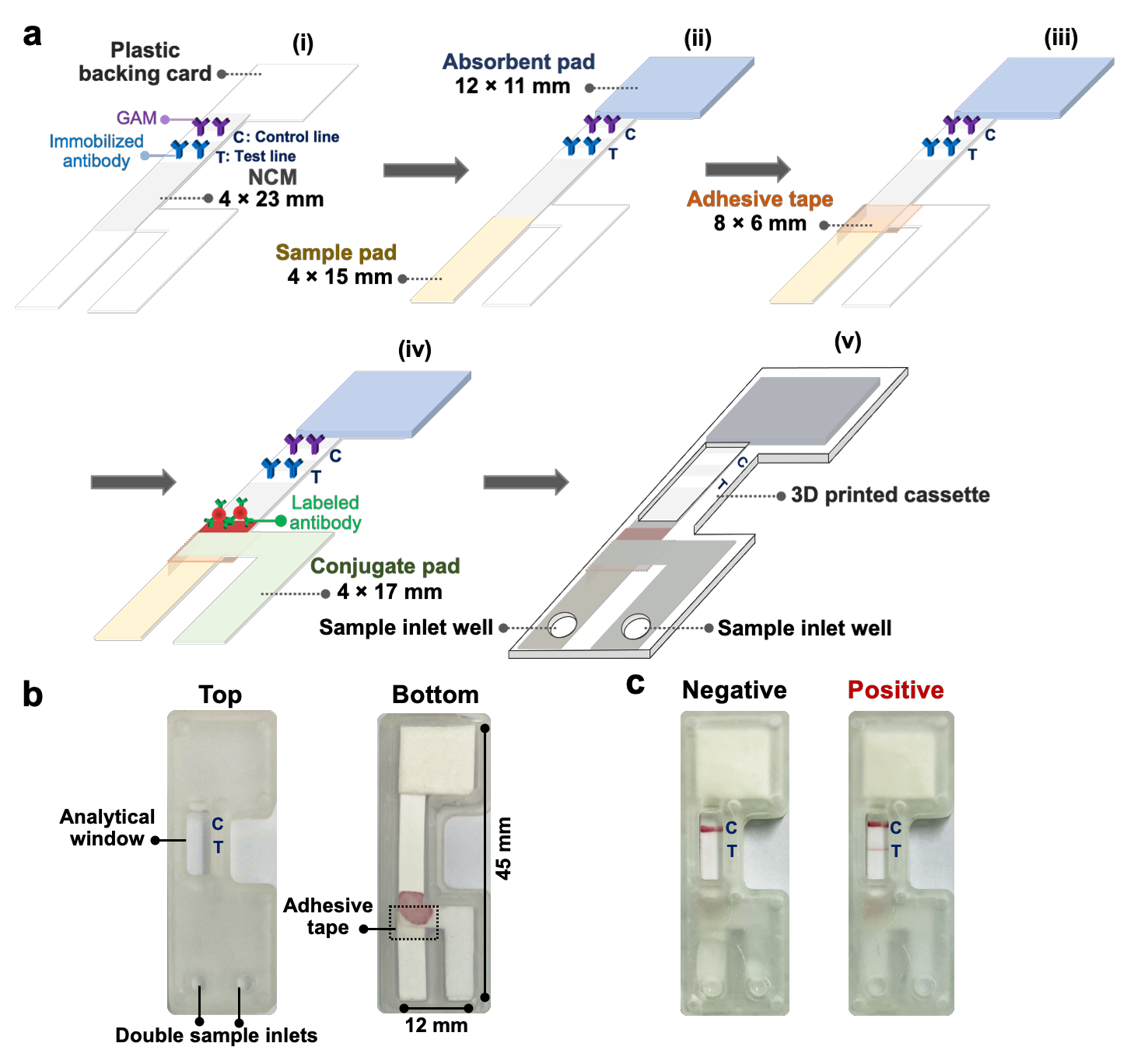


**Figure S4** a) Fabrication procedure of the independent double-sample inlet device: (i) preparation and attachment of the NCM on the plastic backing card, (ii) attachment of absorbent and sample pads, (iii) attachment of the adhesive tape, (iv) preparation and attachment of the conjugate pad and (iv) placement of the device into the 3D printed cassette; b) top and bottom views of the double-sample inlet device (width of 12 mm and length of 45 mm) inside the 3D printed cassette comprising double-sample inlet wells and an analytical window; c) representative data of negative and positive (20 ng/mL of ferritin) results

**References**

1 Mao, X., Du, T.-E., Meng, L. & Song, T. Novel gold nanoparticle trimer reporter probe combined with dry-reagent cotton thread immunoassay device for rapid human ferritin test. *Anal. Chim. Acta* **889**, 172-178, doi:<https://doi.org/10.1016/j.aca.2015.06.031> (2015).

2 Meng, L.-L., Song, T.-T. & Mao, X. Novel immunochromatographic assay on cotton thread based on carbon nanotubes reporter probe. *Talanta* **167**, 379-384, doi:<https://doi.org/10.1016/j.talanta.2017.02.023> (2017).

3 Srinivasan, B. *et al.* ironPhone: Mobile device-coupled point-of-care diagnostics for assessment of iron status by quantification of serum ferritin. *Biosens. Bioelectron.* **99**, 115-121, doi:<https://doi.org/10.1016/j.bios.2017.07.038> (2018).

4 He, Q. *et al.* Lateral Flow Immunosensor for Ferritin Based on Dual Signal-Amplified Strategy by Rhodium Nanoparticles. *ACS Appl. Bio Mater.* **3**, 8849-8856, doi:10.1021/acsabm.0c01169 (2020).

5 Preechakasedkit, P., Ngamrojanavanich, N., Khongchareonporn, N. & Chailapakul, O. Novel ractopamine-protein carrier conjugation and its application to the lateral flow strip test for ractopamine detection in animal feed. *J. Zhejiang Univ. Sci. B* **20**, 193-204, doi:10.1631/jzus.B1800112 (2019).
